# The female to male calf sex ratio is associated with the number of services to achieve a calf and parity of lactating dairy cows

**DOI:** 10.1093/tas/txac080

**Published:** 2022-06-14

**Authors:** Andrew J Mendes, Michael R Murphy, David P Casper, Peter S Erickson

**Affiliations:** University of New Hampshire, Durham, NH 03824, USA; Department of Animal Sciences, University of Illinois, Urbana, IL 61801, USA; Casper’s Calf Ranch, Freeport, IL 61032, USA; Department of Animal Science, North Carolina Agricultural and Technical State University, Greensboro, NC 27411, USA; Department of Agriculture, Nutrition, and Food Systems, University of New Hampshire, Durham, NH 03824, USA

**Keywords:** parity, services per calving, secondary sex ratio

## Abstract

Commercial dairy producers may get frustrated by the lower ratio of female to male calves born because female calves are more valuable than bull calves. Our objective was to determine if parity or stage of lactation at the time of breeding, using conventional semen, influenced the sex of the calf. Data from the University of Illinois and the University of New Hampshire dairy herds were collected and summarized for calf sex, the number of services to achieve a calf and the lactation number when conception of that calf occurred. Logistical regression procedures were used to analyze the dataset via version 9.4 of SAS. The final dataset contained 2,987 calvings, which consisted of 1,406 females and 1,581 males (47.1% and 52.9% for females and males, respectively). The frequency distribution of the number of services to achieve a calf was highest for the first service and progressively declined with increasing services (52.06%, 21.66%, 10.75%, 6.66%, 4.22%, and 4.65% for 1 to 6 services, respectively). The frequency distribution of calvings by lactation number was greatest for first lactation cows becoming pregnant with their second calf and declined with increasing parity (35.49%, 28.22%, 17.01%, 9.61%, 5.02%, 2.51%, 1.14%, 0.70%, and 0.30% for lactation numbers 1 to 9, respectively). Logistic stepwise regression indicated that the number of services to achieve a calf was significant in predicting the ratio of female to male calves. Calculation of odds ratios indicated that as the lactation number increased the likelihood of getting a bull calf decreased. Parity, services, and parity by services interaction were significant for cows having a greater number of parities and cows with a greater number of services yielding more heifer calves. However, an interaction occurred where cows with greater number of services along with greater parities more likely to have a bull calf. These data provide evidence that increasing the number of services to achieve a calf and increasing age of the cow increased the probability of a heifer calf being born. These data indicate that cows with greater parties (lesser cull rate) are more likely to produce heifer calves.

## INTRODUCTION

Darwin suggested that some animal species can exhibit significant shifts in the proportions of male and female offspring that are born, though the conditions and mechanisms that cause these changes are unclear (Rosenfeld and Roberts, 2004). Trivers and Willard (1973) noted that in polygynous species, a small proportion of males, typically the larger and more aggressive ones, share most of the lifetime reproductive success. Lower-ranking males often sire no offspring at all. However, a majority of females, regardless of social ranking or body condition, will be impregnated by that select group of males.

The hypothesis suggested by Trivers and Willard (1973) states that females in the best body condition would tend to produce offspring of the gender that favors the sex of greater variance, namely, males. The male offspring would benefit from greater parental investment and most likely join the elite group of breeding males as adults. As a consequence, females are likely to pass their genes to more of their offspring’s progeny. Conversely, females lower in the social structure or in poorer body condition would invest more in female progeny because their daughters, rather than their sons, are likely to have greater lifetime reproductive success (Rosenfeld and Roberts, 2004). Hossein-Zadeh (2012) observed that younger cows had a greater chance of producing a bull calf than a heifer calf. Possibly, these cows were in better body condition than older, likely higher producing cows. However, Roche et al. (2006a, b) observed no effect of parity on calf sex and Berry and Cromie (2007) reported that older cows were more likely to give birth to male calves compared to younger cows.

Gray and Hurt (1979) evaluated calving data from 1,046 Holstein cows and 4,275 parities from Cornell University. They observed that the average over five parities was 51.97% males. In their data, only the third parity was more likely to produce a heifer calf. Powell et al. (1975) and Berry et al. (2011) found similar ratios of male calves (52.9% males and 51.2% males respectively). Foote (1977), based on data from 5,500 cows receiving 4 or more inseminations, reported no effect of number of artificial inseminations (AI) on calf sex ratio. Meier et al. (2010) observed an increase in the odds of producing a male calf if lactating cows had lower milk fat to protein ratio before conception, were in lower body condition pre-calving or gained condition from calving to the breeding period. Roche et al. (2006a, b) reported similar findings; therefore, it can be postulated that longer-term energy balance rather than short-term energy status influences calf sex ratio. Energy balance changes over the course of lactation, implying that calf gender may be influenced by when the cow conceives. Therefore, current data based on parity and secondary sex ratio are inconclusive and there are scant data regarding number of inseminations and secondary sex ratio.

The objective of this study was to determine if parity or AI service number at the time of breeding lactating Holstein cows influenced the sex of their next calf. Our hypothesis was that parity and AI service number affected the proportions of female and male calves in dairy cows bred with conventional semen.

## MATERIALS AND METHODS

In this retrospective study, dairy herd improvement data were collected from the University of New Hampshire and the University of Illinois research dairy farms (1980–2005) in an observational study with the outcome being either a bull or a heifer calf. Both farms utilized data from their Holstein herds. The University of New Hampshire herd was housed in a tiestall, whereas the University of Illinois herd was housed in both tiestall and freestall facilities. Herd averages over this period ranged from 10,000 to 14,000 liters. Cows were used in various nutritional experiments at both locations which likely impacted milk yield. Data collected included calf sex, AI service number, lactation number, and the interaction of AI service number and parity. These variables entered the logistical regression (SAS version 9.4, Cary, NC) to test the probability of a male calf being born. Stepwise selection was used with a select entry value of 0.3 and a select stay value of 0.35 was used. The Hosmer and Lemeshow goodness of fit test was requested by using the lackfit option in SAS. Eight observations were removed from the data set, 6 due to > 9 services, and 2 due to lactation number > 9. The final data set contained 2,987 (2429 from the University of Illinois and 558 from the University of New Hampshire) calving events including 1,406 females and 1,581 males. The model used was binary as the calf would be either male or female. Cows likely were used more than once but were considered an individual variable as parity would have changed. This may have led to some confounding of the data.

Beginning in 1990, the University of New Hampshire herd began synchronizing estrus. Briefly, the herd has a 50-d voluntary waiting period (VWP). On the first Monday after day 50, an injection of gonadotropin releasing hormone (GNRH) followed by an injection of dinoprost tromethomine (Lutalyse, Zoetis, Kalamazoo, MI) 7 d later and an injection of GNRH 2 d later. Cows were bred 18–24 h after the second GNRH injection. Beginning in 2003, the University of Illinois began using a synchronization program. In that breeding program, lutalyse was given on Wednesday followed by another lutalyse injection 14 d later. After 12 d, an injection of GNRH was given followed by an injection of lutalyse a week later and GNRH 2 d later and breeding 18–24 h later. The earliest first service breeding was at 60 d in milk. All cows were bred to Holstein sires and no cows were bred using sex-sorted semen. All cows were artificially inseminated; no natural mating occurred.

## RESULTS AND DISCUSSION

The frequency distribution and the odds ratio are presented in Table 1. The percentage of cows that calved after becoming pregnant at first service (and included in the dataset) was (1,555/2,987) or 52.1%. The percentage of cows that calved after becoming pregnant at sixth service (and included in the dataset) was (139/2987) or 4.7%. The odds ratio of a bull calf being born was greater if the cow conceived on the first or second service. The odds ratio of a cow likely delivering a heifer was greater when confirmed pregnancy and delivery of a calf occurred as a result of the third, fourth, fifth, or sixth service. As services increased the chances for a heifer calf being born increased (*P *= 0.01).

**Table 1.** Frequency distribution and odds ratio of services per delivered calf from Holstein cows at the Universities of New Hampshire and Illinois

A.I. service^*^	Frequency^†^	Percentage^‡^	Odds ratio estimate^‖^
1	1555	52.06	1.052
2	647	21.66	1.016
3	321	10.75	0.983
4	199	6.66	0.950
5	126	4.22	0.918
6	139	4.65	0.887

Number of artificial insemination services.

Number of calvings in this dataset that calved at a given service.

Percentage of total calvings (2,987).

Odds ratio of male to female calves; >1 = increased chance of a male; <1 = increased chance of a female (*P *= 0.01).

The frequency distribution and odds ratio of calvings by parity are presented in Table 2. The majority of the cows evaluated in this observational study were of first parity (35.5%). The odds ratio of a bull calf tended to be more likely from cows in parity 1, 2, and 3, whereas mature cows (parity 4–9) tended to be more likely to produce a heifer (*P *= 0.08). Overall, regardless of the number of inseminations, younger cows (parity 1 to 3) tended to produce bull calves while parity 4 and greater cows tended to produce more heifer calves (*P* = 0.08). Our data support those of Hossein-Zadeh (2012) who observed that the odds ratio decreased from 1.11 for parity 1 to 1.03 for parity 3. However, our data do not concur with the results of Roche et al. (2006a, b) in regard to parity. They observed no effect of parity on calf sex. Berry and Cromie (2007) reported that older cows were more likely to give birth to a bull, whereas younger cows were more likely to give birth to a heifer. If it is assumed that the greater parity cows used in our experiment were older, these results are incongruous. Hossein-Zadeh (2012) also found that there was a 25.5% chance of delivering a male calf if the first calf was male and a 12.7% chance of having a male calf birth on the third delivery. Roche et al. (2006a) reported the same effect of previous birth sex on the incidence of repeating that sex. Hossein-Zadeh (2012) also observed that seasonality had an effect on the incidence of male calves. More bulls were born in Spring, whereas more heifers were born in Summer. Likewise, Roche et al. (2006a) indicated that there was a one-percentage unit increase in the chance of a bull calf being born for every 1 °C increase in average maximum air temperature (average temperature was 18.3 °C). In our experiment, we were not able to evaluate seasonality effects on sex of subsequent calves.

**Table 2.** Frequency distribution and odds ratio of calvings by lactation number from Holstein cows at the Universities of New Hampshire and Illinois

Lactation^*^	Frequency^†^	Percentage^‡^	Odds ratio estimate^‖^
1	1060	35.49	1.080
2	843	28.22	1.044
3	508	17.01	1.009
4	287	9.61	0.975
5	150	5.02	0.943
6	75	2.51	0.911
7	34	1.14	0.881
8	21	0.70	0.851
9	9	0.30	0.823

Lactation number.

Number of cows in the dataset that calved and associated lactation number.

Percentage of total calvings in this dataset (2,987 calvings).

Odds ratio of male to female calves; >1 = increased chance of a male; <1 = increased chance of a female (*P *= 0.08).

Analysis of maximum likelihood estimates is presented in Table 3. Results were similar between both university farms. However, parity (*P* = 0.04), number of services (*P < *0.03), and parity by service interaction (*P <* 0.03) all impacted calf sex. Cows with greater parity and cows with more services were more likely to have a heifer calf, whereas greater parity cows with more services were more likely to have a bull. These data do not support the results of Foote (1977) who observed no effect of number of inseminations on calf sex ratio, but data from the first three inseminations were not used in his calculations. Our data support the results of Roche et al (2006b) where they observed that cows that lost less body condition score or maintained BW were most likely to have a bull. First lactation cows would likely lose less body condition than cows with greater parity as they are still partitioning nutrients toward growth. We did not measure body condition or BW in this experiment. It is likely that as cows reach maturity body condition score would likely decrease more from birth of the current calf to conception of the next calf, supporting the Trivers–Willard hypothesis where dams with limited resources would be more likely to invest in the more reproductively stable sex, whereas dams in better physiological condition would be advantaged by investing in the more variable sex (Roche et al., 2006b). Therefore, cows in good physiological condition are more likely to produce a bull than a heifer because at the end of parental investment the bull is expected to out-reproduce its sibling heifers (Roche et al.,2006b).

**Table 3.** Analysis of maximum likelihood estimates to produce heifer calves from Holstein cows at the Universities of New Hampshire and Illinois

Variable	df	Estimate	SE^*^	Wald χ^2^	*P* > χ^2^
Intercept	1	−0.067	0.168	0.156	0.693
University^†^	1	−0.056	0.103	0.301	0.583
Parity^‡^	1	0.084	0.041	4.209	0.040
AI^‖^	1	0.111	0.050	4.832	0.028
Parity × AI^$^	1	−0.034	0.016	4.802	0.028

SE = standard error.

Refers to data collected from the University of New Hampshire and the University of Illinois.

Effects of cow parity.

Refers to the number of artificial inseminations.

Refers to the interaction of parity and artificial inseminations.

Berry and Cromie (2007) compared AI cows to cows naturally mated and reported a greater frequency of male calves born if the cow was bred AI. Xu et al. (2000) observed the rate of male calves born in New Zealand and found that there was an increase in male calves born from frozen semen compared to the use of fresh semen. These data support the observation of Berry and Cromie (2007) that natural mating resulted in more heifer calves being born. Xu et al., (2000) postulate that freezing could have affected the survival of some of the X-chromosome bearing sperm.

Dallago et al. (2021) indicated that if dairy cow longevity is increased, involuntary culling would be reduced because of lowered health costs and greater lifetime profitability. Our data suggest that greater longevity may also be associated with a greater likelihood of more heifer calves being produced per cow. Under typical dairy farm situations, it is recommended that cows give birth every 12 to 13 mo. Our study did not evaluate age per se, but parity and number of inseminations. In most dairy operations, parity and age are correlated closely and our results indicated that older cows would likely produce more heifer calves than bull calves.

The frequency of number of services to achieve a delivered calf was highest for the first service and declined rapidly with increasing services. Frequency distribution of calvings by lactation number was highest for first lactation cows and declined with increasing lactation number. These data indicated that the likelihood of a bull calf was greater for younger cows and cows with fewer services per calf.

To the best of our knowledge, the potential effects of level of milk production on secondary sex ratio have not been evaluated. We hypothesize that higher-producing cows would likely have more heifer calves, possibly because they would likely lose more BW after parturition than lower-producing cows. Moreover, higher-producing cows would likely remain in the herd longer and cows with greater parities produced more heifer calves. These data suggest that increased herd longevity could possibly also increase the proportion of heifer calf births.
